# Impact of Sequential Passaging on Protein Expression of *E. coli* Using Proteomics Analysis

**DOI:** 10.1155/2020/2716202

**Published:** 2020-07-30

**Authors:** Mohammed S. Alhajouj, Ghadah S. Alsharif, Ahmed A. Mirza

**Affiliations:** Department of Medical Laboratory Technology, Faculty of Applied Medical Sciences, King Abdulaziz University, P.O. Box 80324, Jeddah 21859, Saudi Arabia

## Abstract

Urinary tract infection (UTI) is one of the most prevalent bacterial infections in the world affecting the bladder and the kidney. *Escherichia coli* (*E. coli*) is the main causative agent of 80–90% of community-acquired UTIs, about 40% of nosocomial UTIs, and 25% of recurrent UTIs. The field of proteomics has emerged as a great tool to analyze expressed proteins to identify possible biomarkers associated with many pathological states and, to the same extent, those associated with bacterial pathogenesis and their ability to cause recurrent infections. Here, in a descriptive cross-sectional pilot study, we employed proteomic techniques to investigate the effects of environmental stress on protein profiles of *E. coli* simulated by sequential passaging of samples from patients with UTIs to screen for unique proteins that arise under stressful environment and could aid in the early detection of UTIs. Four urine samples were collected from individuals with recurrent UTI and sequentially subcultured; protein samples were extracted from bacterial pellets and analyzed using 2-dimensional gel electrophoresis (2DGE). Protein spots of interest arising from changes in the protein profile were analyzed using liquid chromatography-mass spectrometry (LC-MS/MS) and matched against known databases to identify related proteins. We identified ATPB_ECOBW, ASPA ECOLI, DPS ECOL6, and DCEB ECOLI as proteins associated with higher passaging. We concluded that passaging resulted in identifiable changes in the protein profile of *E. coli*, namely, proteins that are associated with survival and possible adaptation of bacteria, suggestive of factors contributing to antibiotic resistance and recurrent UTIs. Furthermore, our method could be further used to identify indicator-protein candidates that could be a part of a growing protein database to diagnose and identify causative agents in UTIs.

## 1. Introduction

Urinary tract infection (UTI) is the second most prevalent infection in humans causing inflammation of the urinary system encompassing the ureters, bladder, urethra, and kidneys. It is predominantly caused by uropathogenic *Escherichia coli* (*E. coli*) and is regarded amongst the most widespread infections in humans affecting both men and women [1–3]. Although UTIs are mostly uncomplicated in healthy individuals, they pose complications in immunocompromised individuals, sexually active women, individuals with a history of recurrent UTIs, patients under intensive care, and users of condoms and spermicidal sprays [1].

Due to the anatomical structures of the female urinary tract, UTIs are more prevalent in females than males [1]. In fact, 12% of all women develop UTI annually and 50% of female patients under 32 had experienced a minimum of one UTI during their lifetime [4]. Furthermore, 25% of young women who had their first cystitis will be reinfected again within 24 weeks due to the increased rate of recurrent UTIs as compared to primary infections [4–6]. Although bacteria prove to cause about 70–95% of community-acquired infections and 50% of nosocomial infections, *E. coli* is the major causing factor of UTIs responsible for 80–90% of all community-acquired UTIs, 40% of nosocomial UTIs, and 25% of recurrent UTIs [7–9]. Moreover, a higher risk of recurrent UTI affects 20% of hospitalized geriatric patients where *E. coli* cause more than 70% of all cases and is atypical in more than half of the studied subjects [10], increasing the risk of mis- or underdiagnosis.

Uropathogenic *E. coli* (UPEC) is any *E. coli* strain that causes UTIs and is considered the key origin of UTI [7]. Such bacteria utilize adhesions, toxins, and iron acquisition as virulence factors and are released in the human urinary tract to complete its growth and to cause UTI [11]. More importantly, UTIs can cause more serious diseases that extend to kidney failure, should not be underestimated, and should be treated as soon as possible by antibiotics to eliminate the bacteria causing the inflammation. Accordingly, it is imperative and clinically useful to find surrogate biomarkers in the serum or urine for quick identification of recurrence in patients with a first UTI to avoid further complications such as pyelonephritis. Since the origin of *E. coli* is found to be in the bowel with numerous differences in commensal intestinal *E. coli* strains, UPEC isolates need to be studied at a proteomic level.

It is well documented that *E. coli*, or bacteria in general, adapt to changing environmental cues and conditions [12–14]. Previous research suggested that several identifiable proteins play an important role in recurrent infections and can be unique amongst certain bacteria individually or amongst a profile of proteins that could act as an identifiable fingerprint. Accordingly, bacterial protein profiles can possibly be unique biomarker fingerprints of infections in general and more specifically with those associated with UTIs. Moreover, urinary biomarkers for lower urinary tract diseases have been widely discussed for years. The first possible biomarkers found in recurrent UTI were serum antibodies. In a previous study, women patients with recurrent UTI who had completed antibiotic treatment had significantly higher levels of serum antibody immunoglobulin (Ig)G, IgM, and IgA than those found in healthy controls [15]. However, there are many factors that shape the protein profile of bacteria such as the environmental stress of the host's immune system and the length of infection. Accordingly, bacteria evolved to adapt to the varying and possibly extreme environment surrounding it such as antimicrobial agents, nutritional limitations, heat, and oxidative damage, by launching an effective and evasive biological response [16]. While bacteria have several mechanisms that can be used to face environmental challenges *in vivo*, the common step in its response is receiving the environmental stressor through molecular signal transduction soliciting a transcriptional response and leading to the production of the necessary proteins for adaptation [17]; however, *in vitro*, a common method to induce such changes is sequential passaging.

Several studies investigated the effects of sequential passaging on multiple organisms and showed both reduction and amplification of adaptation mechanisms. In fact, one study repeated *in vitro* sequential passaging of *Salmonella enterica* for up to 270 times to elucidate the mechanism of genome reduction as an evolutionary model for metabolic regulation and adaptation [18]. Similarly, adaptation to specific environments was demonstrated by several sequential passages over a six-week period in *Staphylococcus aureus*, resulting in accessory gene regulatory (*agr*) operon variants and leading to genotypic and phenotypic changes such as the loss of the characteristic beta-hemolytic ability of *S. aureus* [19]. On the contrary, *in vivo* sequential passaging of *E. coli* K1, associated with life-threatening gastrointestinal tract infections in neonates, resulted in a three-time more aggressive and lethal variant strain. Interestingly, the factors contributing to the more ferocious variant of the original isolate are not related to the usual virulence factors associated with invasiveness; instead, they were all single-nucleotide point polymorphisms in genes associated with metabolic pathways of the organism [20].

Finally, proteomics technology has been used to study bacterial pathogenesis. Although sodium dodecyl sulfate-polyacrylamide gel electrophoresis (SDS-PAGE) is mainly used to separate proteins according to their mass and is the default initial screen of change in protein expression, it offers little insight of the complexity of changes in the expression profile. On the other hand, 2DGE offers a far more powerful tool to meticulously evaluate and pinpoint unique changes in the protein expression profile of any organism, and when coupled with mass spectrometry, it becomes a powerful tool to identify exact proteins according to precise molecular weight [21, 22]. In our study, we aimed to investigate the changes in the protein profile of *E. coli* bacteria forced into adaptation under the stressful environment of sequential passaging and to take the first step to identify unique proteins that could possibly be used in the future for the early diagnosis of UTIs caused by *E. coli* [23].

## 2. Materials and Methods

### 2.1. Source of *E. coli* Isolates

Four anonymous urine samples associated with cases of recurrent UTI were obtained at random for secondary use from routine samples coming to the Department of Medical Microbiology of Aberdeen Hospital, Aberdeen, UK. The samples were not intended for this project specifically and had no clinical or personal identifiers; consent and ethical approval were not required. Bacterial isolates were collected from urine culture and plated onto Colombian agar plates and incubated aerobically at 37°C. All laboratory work was completed at the Proteomics Facility, Aberdeen University, Aberdeen, UK.

### 2.2. Sequential Passage

*E. coli* colonies isolated from original Colombian agar plates were subcultured on new Colombian agar plates and incubated aerobically overnight at 37°C. This step was repeated eight times. Only samples from the first and last subcultures were prepared for proteomics comparison and further analysis.

### 2.3. Microorganism Profiling with MALDI Biotyper

The protocol described by Benjamin et al. was used to identify the species of *E. coli* [24]. Briefly, a single colony was plated on Colombian agar plates and left incubated aerobically overnight at 37°C. To prepare the matrix solution, 1 mL of basic organic solvent (OS) was prepared daily as follows: 500 *μ*L Acetonitrile was added to 475 *μ*L ultrahigh quality (UHQ) water and 25 *μ*L trifluoroacetic acid, and then 10 mg of *α*-cyano-4-hydrocinnamic acid (HCCA) matrix (Bruker, Bremen-Germany) was mixed in the solution. Next, samples for MALDI Biotyper were prepared by transferring 5–10 mg of bacterial mass into a microcentrifuge tube and mixed thoroughly with 300 *μ*L UHQ water. Next, 900 *μ*L of ethanol was added and vortexed thoroughly, centrifuged at a maximum speed of 14,000 rpm for 2 minutes, and then the supernatant was decanted; the tube was recentrifuged a second time and residual ethanol was removed by careful pipetting. Next, 50 *μ*L of 70% formic acid solution in UHQ water and 50 *μ*L of pure acetonitrile were added to the pellet and then carefully mixed, centrifuged at maximum speed for 2 minutes, and then the supernatant was decanted. Next, 1 *μ*L of precipitate was placed onto a MALDI ground steel target plate and left to air-dry. Finally, 1 *μ*L of matrix solution was overlaid directly on the dry bacterial mass, let to dry, and then immediately analyzed. Samples were analyzed using Bruker Daltonik MALDI Biotyper Classification Results (BDMBCR) (Bruker Daltonik, Germany). The MALDI Biotyper identifies microorganisms using Matrix-Assisted Laser Desorption Ionization/Time of Flight (MALDI-TOF) mass spectrometry to determine the unique proteomic fingerprint of an organism. The characteristic spectrum pattern of this proteomic fingerprint is used reliably and accurately to identify microorganisms by matching it against thousands of referenced spectra of a multitude of strains.

### 2.4. Preparation of Protein Extract from Bacterial Cells

The protocol used here was similar to the one described by Smith et al. [25]. Briefly, *E. coli* colonies were scraped from the surface of several Colombian agar plates, washed, and resuspended in phosphate-buffered saline (PBS), and optical density (OD) was determined at 600 nm to accurately calculate the bacterial cell mass and lysis buffer volume needed thereafter. 100 *μ*L of 2D lysis buffer (Bio-Rad, Germany) for every 0.1 OD_600_ was added to the bacterial cell pellet, gently resuspended, left on ice for 10 minutes, and then sonicated by Sonic Dismembrator FB-505 (Fisher Scientific, USA) 3 times for 30-second-On-Off intervals at level 7 while on the ice. Finally, lysates were centrifuged at 13,000 rpm for 5 minutes to remove cell debris and the supernatant was stored at −20°C for subsequent analysis.

### 2.5. Analysis of Bacterial Proteins by 2DGE

Two-dimensional gel electrophoresis was completed according to the manufacturer's instructions. In brief, the first phase of the protocol, isoelectric focusing, was completed as follows: 125 *μ*L of the bacterial lysate was uniformly spread in the slots of the Immobline® DryStrip (GE Healthcare, Sweden) immobilized pH gradient (IPG) gel in a reswelling tray and carefully spread. Owing to its sufficient resolution, only pH range 4–7 was used as suggested by previous research [26]. Extra care was taken to assure that no air bubbles were trapped between the sample and strip. Mineral oil was overlaid on the IPG strips and trays were covered and left to stand overnight to rehydrate the gel strips. Next, the rehydrated IPG strips were removed from the reswelling tray and placed onto the strip aligner of a Multiphor II flat-bed electrophoresis system (GE Healthcare, Sweden). The IPG gel strips were connected to the electrodes and filter wicks were cut to 11 cm, soaked in MilliQ water, and placed at the top surface of the IPG strips. Next, electrode bars were placed over the electrode filters and the leads were connected to the EPS 3,500 XL Power Supply (GE Healthcare, Sweden) and set to run at 200 volts for one minute, ramped up to 3,500 volts over 90 minutes, and then ran at 3,500 volts for 65 minutes. The IPG gel strips were removed from the Multiphor II apparatus, excess oil was removed by blotting on filter paper, placed in sealed plastic tubes, and stored at −20°C until further processing. The second phase of the protocol, second dimension gel electrophoresis, was completed according to the manufacturer's protocol as follows: IPG strips were brought up to room temperatures and equilibrated first in IPG equilibration buffer containing 10 mg/mL dithiothreitol (DTT) for 30 minutes and then in equilibration buffer containing 25 mg/mL iodoacetamide for 30 minutes on a shaker in both steps. The strips were then immersed in MilliQ water and then let dry for 10 minutes. Next, Invitrogen precast commercial gels (Bio-Rad, Germany) were assembled to the SE250 electrophoresis units (GE Healthcare, Sweden). Inner and outer chambers were filled with electrophoresis running buffer (Trizma base 3 g, glycine 14.4 g, and SDS 1 g) and the gels were electrophoresed initially at 75 volts for one hour and then 150 volts for two and a half hours. Next, gels were placed in the fixative solution overnight (50% ethanol, 2% mL phosphoric acid in MilliQ water) on a shaker. Finally, gels were washed in MilliQ water three times for 30 minutes each and stained using 0.2 g Coomassie Brilliant Blue G250 powder (Bio-Rad, Germany) in equilibration buffer (34 g of ammonium sulfate, 68 mL of methanol, 4 mL of phosphoric acid, and 100 mL of water) for up to 4 days on a shaker. Once optimal staining was achieved, gels were scanned by Image Scanner™ III (GE Healthcare, Sweden) for further processing.

### 2.6. Comparative Analyses of Protein Profiles

The digitized images of stained gels were transferred to Progenesis SameSpots software version 4 (Nonlinear Dynamics, UK) for comparative analysis between samples. Briefly explained, based on the least significant difference between the images, the software aligns gels to detect spots based on analysis of variance (ANOVA) statistical test. Only spots with *P* values ≤0.05 were significant and to be investigated. To control for possible variations due to protein amount loaded on the 2D gel, despite our best effort to equalize it based on our initial SDS-PAGE gel run, the SameSpots software uses internal algorithms that normalize differences to a reference and then compensate for any possible errors. The program analyzes the least different image from the set of images and set the average of all spot intensities therein as ratio distribution around 1. To accommodate changes due to up- and downregulation of protein spots, the ratios are calculated on a log scale and intensities vary around a zero average. The program then treats all images equally but sets their averages relevant to the reference gel. Accordingly, internal averages of other gels in the set will vary above or below zero when compared to the reference and a correction factor is used to adjust the intensities and eliminate variations due to loading errors or image processing. Finally, the program assigns a number to each spot of possible interest according to the Progenesis SameSpot software protocol. The slight modification in this experiment, however, is rather than having the program determine the reference gel as described above; the two gels of the prepassaging and the postpassaging cultures formed a 2-gel set where the prepassaging gel image was set as a reference and the postpassaging as a test. Although many proteins exhibited up- or downregulation, only four proteins were investigated; those selected spots were clearer, showed strong upregulation patterns, and were easily excisable. A 3D montage of gel images of the differentially expressed proteins was created by the software to better illustrate the change in the expression.

### 2.7. Protein Identification Using Liquid Chromatography-Mass Spectroscopy (LC-MS/MS)

The Dionex U3000 Liquid Chromatography System and the Bruker Daltonics HCT Ion Trap Mass Spectrometer were used for protein identification. First, spots of interest were excised from the dried gel using a clean scalpel and rehydrated in UHQ water for 30 minutes. DTT was then added to samples and incubated at 60°C for 20 minutes, S-alkylated with iodoacetamide at 25°C for 10 minutes, and then digested with trypsin for 8 hours at 37°C. Samples were finally dried using a rotary evaporator and then dissolved in 10 *μ*L 0.1% formic acid in water for LC-MS/MS analysis. Next, samples were placed into a 96-well plate and covered with reusable silicone sealing mat, vortexed thoroughly, and centrifuged for 1 minute; the reusable silicone sealing mat was then replaced with sealing film. Samples were then loaded onto the LC system for analysis. Finally, results were analyzed using the Mascot program against a database and identification based on ion scores as a nonprobabilistic basis for ranking possible protein hits.

## 3. Results

### 3.1. Bacterial Strains

All bacterial species, genus, and strains of used samples serially numbered according to their acquisition (7, 15, 274, and 515) were verified at the fourth passaging of the original culture using MALDI-TOF. Accordingly, the causative agents for the UTIs in the selected samples were identified using BDMBCR to be one of two strains of *E. coli*, *Escherichia coli* DH5alpha BRL, or *Escherichia coli* MB11464_1 CHB (Table 1).

### 3.2. Protein Identification

**Figure 1:**
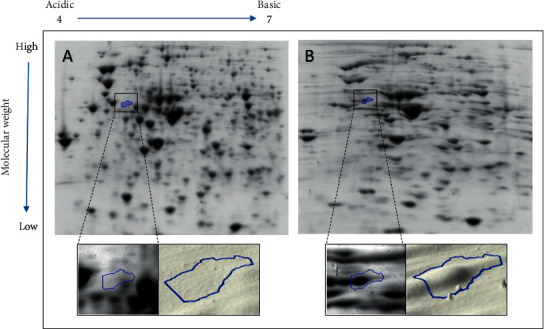
Comparative analyses of protein profiles of sample number 7.

**Figure 2:**
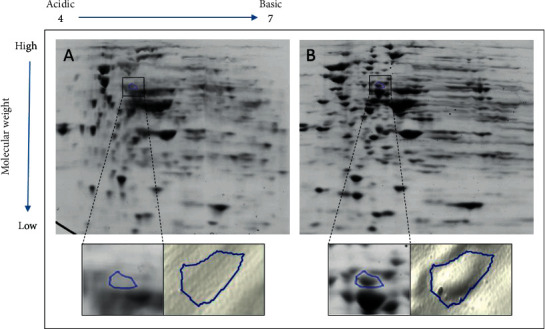
Comparative analyses of protein profiles of sample number 15.

**Figure 3:**
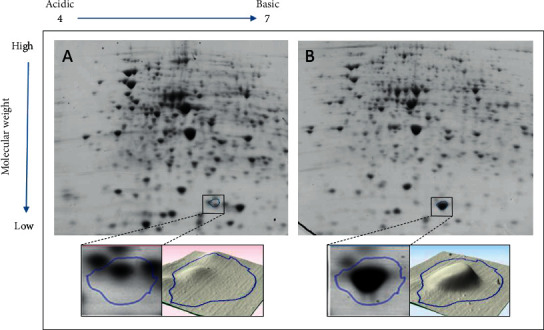
Comparative analyses of protein profiles of sample number 274.

**Figure 4:**
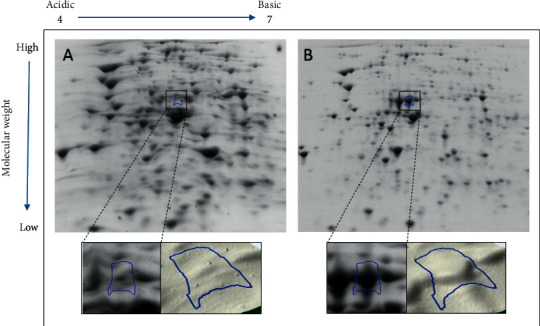
Comparative analyses of protein profiles of sample number 515.

Four spots were selected from the passaged gels of all samples, namely, spot number 1131 from sample 7, spot number 756 from sample 15, spot number 1924 from sample 274, and spot number 1789 from sample 515. Figures 1–4 illustrate the positions of the four spots of interest in the four selected samples according to molecular weight and isoelectric point. SameSpots software generated 3D montage to visualize the differences amongst the same spots in original and passaged gels. Differences in protein expression pattern in samples 7, 15, 274, and 515 (Figures 1–4, respectively) are clear between the first passaged samples (A) and the last passaged sample (B). 3D rendering montage shows the change in intensity of the selected spot (lower panel).

Mascot scores for spots were matched with proteins in the Swiss-Prot database. According to the UniProt database, four proteins were identified from the excised spots of interest, namely, ATPB_ECOBW, ASPA ECOLI, DPS ECOL6, and DCEB ECOLI (Table 2).

## 4. Discussion

Several studies used proteomics as a means to evaluate protein adaptation and alteration and more specifically to develop applications to speciate and differentiate bacteria according to synthesized proteins [1]. Although comparative genomics provides important details about the bacterial development, no study yet can present a full description of host-interaction induced bacterial gene expression. In this research, we simulated the harsh environment surrounding the bacteria using sequential passaging to screen for stress-activated genes. Our goal is to eventually identify a unique panel of protein candidates for the diagnosis of recurrent UTIs caused by *E. coli*. Many proteins were up- and downregulated when comparing 2D gels from the initial versus the last subculture samples. We identified ATPB_ECOBW, ASPA ECOLI, DPS ECOL6, and DCEB ECOLI as significantly upregulated proteins.

ATPB_ECOBW is present in the cell's inner and peripheral membrane and is a part of the ATP synthase apparatus (ATPase alpha/beta chains family) and produces ATP from ADP powered by a proton gradient across the membrane. The catalytic sites are housed in the beta subunits, which catalyzes the transfer of protons from one side of a membrane to the other. The molecular function of ATPB_ECOBW is interacting selectively and noncovalently with ADP to power ATP synthesis by transporting protons across the membrane to generate an electrochemical gradient (proton-motive force) [27]. We can speculate here that the upregulation of this protein was brought on by an increased demand for ATP inside the cell under the environmental stress due to subculturing. Thus, making ATPB_ECOBW a possible indicator of a stressful environment surrounding *E. coli* requiring adaptation. Next, ASPA ECOLI belongs to the class II fumarase/aspartase family, is found in the cytosol and membrane of *E. coli*, and catalyzes the reaction turning L-aspartate to fumarate, regulating a key compound in the tricarboxylic acid cycle needed for energy production. Although finding such protein that functions as a regulator of the TCA cycle upregulated under stressful environment is interesting on its own, it is even more interesting to know that the ASPA ECOLI also upregulates amino acid synthesis and pathways resulting in the synthesis of organic acids baring one or more amino substituents [28]. Again, one can speculate that, under environmental stress, ASPA ECOLI is upregulating energy recruitment and synthesis of essential amino acids used for the *de novo* synthesis of proteins that are crucial for adaptation [2], coinciding with the upregulation of ATPB_ECOBW. And so, ASPA ECOLI could be a possible indicator of a stressful environment. Next, DPS ECOL6, a member of the DNA-binding protein from starved cells (DPS) family, is present in the nucleoid and the cytoplasm and protects the cell DNA from oxidative damage. It does so by sequestering intracellular Fe^2+^ ion, storing it in the form of Fe^3+^ oxyhydroxide mineral, and can then be released after reduction according to the following reaction: 2Fe^2+^ + H_2_O_2_ + 2H^+^⟶2Fe^3+^ + 2H_2_O. Furthermore, DPS protects the cell from several stressors, namely, thermal stress, acid and base imbalance, iron and copper toxicity, and even UV and gamma irradiation. It does so by structure-specific DNA noncovalent binding in the presence of ferric iron, Fe (III), forming a highly ordered and stable double-stranded-DNA cocrystal to condense DNA [29]. Here, we identified DPS ECOL6 as the second most upregulated protein amongst the selected ones to be investigated. Not surprisingly, DPS plays a major role in the survival of bacteria in a stressful environment and hence its name. Like the two previously discussed proteins, DPS ECOL6 could yet be another candidate for key indicators of *E. coli* bacteria under a stressful environment. Finally, DCEB ECOLI, glutamate decarboxylase beta, is present in the bacterial cell membrane and cytoplasm; it decarboxylates L-glutamate to 4-aminobutanoate as a part of the glutamate metabolism pathway and is involved in pyridoxal phosphate binding as well. Furthermore, DECB ECOLI also plays a major survival role in acid resistance when the pH decreases in the environment around the bacteria [30]. Although out of the four proteins investigated here, DCEB ECOLI was the least upregulated with only a 1.5-fold increase, it certainly plays a role in a harsh environment. Here, we suggest that as bacteria produce CO_2_ as they increase in number during subculturing, which eventually turns to carbonic acid as it dissolves in water and decreases pH, DCEB ECOLI is upregulated. Perhaps, DCEB ECOLI would have been more upregulated had subcultures remained for longer periods turning the media more acidic. Regardless of the level of upregulation, DCEB ECOLI is another strong candidate for *E. coli* adaptation under the stressful environment of subculturing.

Our research was able to induce possible adaptation proteins' production through sequential passaging of bacteria, possibly similar to that during UTI. The identified proteins ATPB_ECOBW, ASPA ECOLI, DPS ECOL6, and DCEB ECOLI are closely related regulating enzymes involved in the TCA cycle or cellular receptors that aid in the adaptation and persistence of *E. coli* under stressful environment. It is important to note that ASPA ECOLI and DPS ECOL6 were the most upregulated and then closely followed by ATPB_ECOBW, suggestive of a collaborative network of proteins upregulated under stress to ensure the adaptation of the bacteria in conditions brought on by subculturing. Furthermore, we suggest that the identified proteins could all be possible adaptation factors that might play a role in the ferocity of the bacteria during infections and their recurrence.

Since this research was only a pilot study, further investigation using artificial urine liquid media, simulating the reduction of carbohydrates, lower pH, and increased protein usually observed in UTI urine samples can further confirm the role of the proteins identified here. Furthermore, we suggest that samples from recurrent and atypical UTIs should be compared to similarly passaged reference bacteria to further validate this method. Also, the possibility that the identified proteins operate individually or in conjunction as a part of an “adaptation proteome” needs to be further explored by examining their specific or combined expression before and after induced environmental stress. Lastly, the presence of the identified proteins needs to be verified in fresh urine samples collected from patients with recurrent UTIs.

## 5. Conclusion

In this study, variations in protein expression were created between the original culture and the last of 8 subcultures of *E. coli* isolated from different UTI patients; we conclude that the effect of passaging was clearly observed on the protein profiles of this common pathogen's adaptation to environmental changes. Furthermore, we screened for and identified four possible proteins that arise in *E. coli* due to adaptation to the stressful environment of sequential passaging. We suggest that further studies are needed to link the role of the identified proteins in adaptation, individually and as a group. We hope that the four identified proteins could be candidates amongst a battery of proteins used as indicators for recurrent UTIs, drug development, or vaccinations in the future.

## Figures and Tables

**Table 1:** Bruker Daltonik MALDI Biotyper results.

Sample	Organism ID	Species results	Score
**7**	*Escherichia coli*	*Escherichia coli* DH5alpha BRL	2.294
**15**	*Escherichia coli*	*Escherichia coli* DH5alpha BRL	2.174
**274**	*Escherichia coli*	*Escherichia coli* MB11464_1 CHB	2.288
**515**	*Escherichia coli*	*Escherichia coli* MB11464_1 CHB	2.103

**Table 2:** The identified proteins from the excised spots of interest.

Gel no.	Spot no.	Protein name	Molecular weight	Fold increase
**7**	1131	Adenosine triphosphate (ATP) synthase subunit beta (ATPB_ECOBW)	50,351 Da	2.6
**15**	756	Aspartate ammonia-lyase (ASPA ECOLI)	52,950 Da	4.3
**274**	1924	DNA protection during starvation protein (DPS ECOL6)	18,742 Da	3.2
**515**	1789	Glutamate decarboxylase beta (DCEB ECOLI)	53,204 Da	1.5

## Data Availability

All data used to support the findings of this study are included within the article.
